# Health literacy, social support, and health activation as determinants of health-promoting behaviors in patients with chronic heart failure: a COM-B model–based path analysis

**DOI:** 10.3389/fpubh.2026.1895437

**Published:** 2026-07-15

**Authors:** Siyu Deng, Meijie Zheng, Wenxiu Liu, Mingjin Jiang, Yanmei Wang

**Affiliations:** 1 School of Nursing, Shanghai University of Traditional Chinese Medicine, Shanghai, China; 2 Department of Social Work, Shanghai Municipal Hospital of Traditional Chinese Medicine, Shanghai University of Traditional Chinese Medicine, Shanghai, China; 3 Department of Nursing, Hebei General Hospital, Shijiazhuang, China; 4 Department of Gynaecology and Obstetrics, Shanghai Municipal Hospital of Traditional Chinese Medicine, Shanghai University of Traditional Chinese Medicine, Shanghai, China

**Keywords:** chronic heart failure, COM-B model, health activation, health literacy, health-promoting behaviors, path analysis, perceived social support

## Abstract

**Background:**

Chronic heart failure (CHF) is a progressive and recurrent condition associated with high hospitalization rates, substantial healthcare burden, and impaired quality of life. Health-promoting behaviors are essential for long-term disease management in patients with CHF, yet their formation and maintenance are often constrained by individual capability, external support, and motivational factors.

**Objective:**

This study aimed to examine the current status and associated factors of health-promoting behaviors among patients with chronic heart failure and to clarify the pathways among related factors.

**Methods:**

A cross-sectional design was used to investigate health-promoting behaviors and their associated factors among patients with CHF. The study was conducted from December 2024 to November 2025 in two tertiary Grade A hospitals in Shanghai. Convenience sampling was used, and 320 questionnaires were distributed; 302 valid questionnaires were included, yielding a valid response rate of 94.38%. The instruments included a general information questionnaire, the Health-Promoting Lifestyle Profile, the Perceived Social Support Scale, the Heart Failure-Specific Health Literacy Scale, and the Health Activation Scale. SPSS version 26.0 was used for descriptive statistics, univariate analysis, and multiple linear regression. AMOS version 23.0 was used for path analysis, and Python version 3.9 was used for random forest-based feature importance ranking.

**Results:**

The total health-promoting behavior score was 126.00 (116.00, 137.00), and the mean item score was 2.42 (2.23, 2.63), indicating an overall moderate level; the largest proportion of patients was classified as moderate (52.32%). Among the dimensions, self-actualization had the highest score, whereas physical activity had the lowest score. Health literacy was at a moderate level, perceived social support was at a high level, and health activation was at a low level. Univariate analysis showed significant differences in health-promoting behavior scores by age, educational level, employment status, marital status, living arrangement, monthly per capita household income, type of medical insurance, disease duration, number of comorbidities, and number of hospitalizations in the past year (*p* < 0.05). Health-promoting behaviors were positively correlated with health literacy, perceived social support, and health activation (*p* < 0.01). Multiple linear regression analysis showed that monthly per capita household income, type of medical insurance, number of comorbidities, number of hospitalizations in the past year, employment status, health activation, perceived social support, and health literacy were independently associated with health-promoting behaviors, jointly explaining 68.7% of the variance. Mediation analysis showed that health activation partially mediated the associations of health literacy and perceived social support with health-promoting behaviors. Nonlinear analysis further suggested that number of hospitalizations in the past year, health activation, perceived social support, and health literacy were key features associated with health-promoting behaviors.

**Conclusion:**

Health literacy, perceived social support, and health activation were key factors associated with health-promoting behaviors among patients with CHF. Health activation served as a significant mediator linking health literacy and perceived social support with health-promoting behaviors. Future interventions should focus on improving health literacy, strengthening social support systems, and enhancing health activation to promote the development and maintenance of health-promoting behaviors among patients with CHF.

## 1 Background

Chronic heart failure (CHF) is a clinical syndrome that commonly represents the terminal stage of various cardiovascular diseases. Its incidence and mortality continue to rise worldwide, imposing a substantial burden on patients, families, and healthcare systems (1–3). According to the China Cardiovascular Health and Disease Report 2023, approximately 330 million people in China are living with cardiovascular disease, including about 8.9 million with heart failure (4). CHF commonly presents with dyspnea, fatigue, and fluid retention, and its clinical course is often prolonged and recurrent, with frequent episodes of exacerbation or decompensation (5). Owing to its high hospitalization rate, high mortality (6), and substantial healthcare costs (7), CHF has become a major public health challenge in China and worldwide. Despite advances in pharmacological and device-based therapies, such as angiotensin receptor–neprilysin inhibitors and implantable cardioverter-defibrillators, the prevention and management of CHF in China remain challenging (8). Current clinical management still largely focuses on relieving acute symptoms and slowing disease progression, whereas behavioral support, continuity of care, and psychosocial support are often underemphasized in long-term, life-course management (9) Consequently, patients with CHF may experience persistent physical and psychological burdens and generally poor quality of life (10).

A growing body of evidence suggests that unhealthy lifestyles, poor treatment adherence, and inadequate self-management skills are key modifiable contributors to recurrent exacerbations, high rehospitalization rates, and increased mortality risk among patients with CHF (5, 11). Therefore, the role of health-promoting behavior (HPB) in CHF management has become increasingly important (12). HPB refers to multidimensional, voluntary, and sustained behaviors through which individuals maintain or improve their health (13). National and international guidelines have increasingly emphasized HPB as a core component of CHF care. The 2021 ESC Guidelines on cardiovascular disease prevention in clinical practice emphasize lifestyle modification and patient education as important components of cardiovascular disease prevention and management (14). Similarly, China’s 14th Five-Year Plan for National Health and the Healthy China 2030 Outline call for the comprehensive implementation of nationwide health promotion initiatives (4, 15). Effective promotion and management of HPB may help prevent adverse cardiovascular events (16) and represents a cost-effective strategy for improving prognosis and reducing readmission rates in patients with CHF (17).

Although the evidence-based value of health-promoting behaviors has been well established, adherence to these behaviors often declines as patients experience the long-term burden of chronic illness. Many patients recognize the importance of healthy behaviors but find it difficult to initiate and maintain them consistently, leading to lower-than-expected engagement in health management. Studies have shown that recurrent exacerbations in patients with chronic heart failure (CHF) are often associated with poor rehabilitation adherence and persistent unhealthy behaviors (18). This gap between knowledge and action suggests that behavioral change among patients with CHF is shaped by multiple interacting factors. Previous studies have identified several factors associated with health-promoting behaviors, including demographic characteristics, such as age and educational attainment; physiological factors, such as cardiac function classification (19); psychological factors; and social support.

The Capability, Opportunity, and Motivation–Behavior model (COM-B), proposed by Michie et al. in 2011, serves as the core theoretical framework of the Behavior Change Wheel (BCW) (20). It proposes that behavior and behavioral change result from the interaction of three components: capability, opportunity, and motivation. Because of its clear structure, concise logic, and strong practical orientation, the COM-B model has been widely applied in health behavior promotion, chronic disease management, and nursing intervention research (21, 22). The model emphasizes that capability, opportunity, and motivation do not operate in isolation; rather, they dynamically interact to shape the formation and maintenance of behavior. When any of these components is insufficient, behavioral change may be impeded. Therefore, health behavior promotion interventions that focus solely on knowledge transmission or guidance for a single behavior are unlikely to achieve sustained effects. Instead, facilitators and barriers to behavioral change should be systematically identified across multiple dimensions.

Based on the above considerations, this study adopts the Capability, Opportunity, and Motivation–Behavior model (COM-B) as its theoretical framework. Using multiple quantitative analytical methods, this study systematically identifies determinants of health-promoting behaviors among patients with CHF across three COM-B dimensions: health literacy as capability, social support as opportunity, and health activation as motivation. It further examines the mediating role of health activation in the associations of health literacy and social support with health-promoting behaviors. By integrating multiple linear regression, observed-variable path analysis, and nonlinear analysis, this study identifies key predictors of health-promoting behaviors and further describes the statistical associations and relative importance of these predictors within the COM-B framework. The findings may provide a theoretical basis and practical guidance for healthcare professionals to identify patients with CHF who are at high risk of insufficient health-promoting behaviors and to develop comprehensive intervention strategies focused on improving health literacy, strengthening social support, and enhancing health activation. These strategies may promote active patient participation in disease management and improve long-term health outcomes.

## 2 Participants and methods

### 2.1 Participants

This cross-sectional study used convenience sampling. From December 2024 to November 2025, patients with chronic heart failure were recruited from the cardiology wards and outpatient clinics of two tertiary hospitals. The inclusion criteria were as follows: ① meeting the diagnostic criteria for CHF (23) and classified as New York Heart Association (NYHA) functional class II–IV; ② having a clinical diagnosis of CHF with stable disease control after receiving standardized heart failure treatment; ③ aged 18 years or older; ④ being conscious, able to communicate effectively, and able to complete the questionnaire independently or with assistance from the investigator; and ⑤ providing informed consent and voluntarily participating in the study. The exclusion criteria were as follows: ① severe failure of major organs; ② a history of psychiatric disorders or cognitive impairment; and ③ major life changes or stressful events within the previous three months, such as bereavement, divorce, unemployment, or bankruptcy.

The sample size was estimated according to Kendall’s principle, which recommends that the sample size for multivariate analysis should be 10–15 times the number of independent variables (24). In this study, the independent variables included general demographic characteristics (13 variables), heart failure-specific health literacy (3 dimensions), perceived social support (3 dimensions), and health activation assessed using the Health activation Measure (3 dimensions), yielding 22 predictor variables in total. Accordingly, the required sample size was estimated to range from 220 to 330 participants. Because an observed-variable path analysis model was also planned, the sample-size requirement for path modeling was further considered. According to commonly used methodological recommendations, a sample size of at least 200 is generally considered acceptable for path models estimated using maximum likelihood (25). Therefore, the final valid sample of 302 participants met the recommended requirements for both multivariable regression and observed-variable path analysis Considering these requirements and an anticipated invalid questionnaire rate of approximately 10%, the planned number of questionnaires to be distributed was 245–367. A total of 320 questionnaires were distributed. After data screening, 302 valid questionnaires were obtained, yielding a valid response rate of 94.38%, which met the sample size requirements for subsequent statistical analyses.

### 2.2 Research tools

#### 2.2.1 General information questionnaire

A researcher-designed general information questionnaire was used to collect participants’ sociodemographic and disease-related information. The questionnaire included sociodemographic variables, including sex, age, height, weight, educational level, employment status, marital status, living arrangement, monthly household income, and type of medical insurance, as well as disease-related variables, including disease duration, number of comorbidities, NYHA functional class, and number of CHF-related hospitalizations in the past year.

#### 2.2.2 Health promoting lifestyle profile-II (HPLP-II)

This study utilized the Health Promotion Lifestyle Profile-II (HPLP-II), developed by Walker (26) et al., for data collection. The scale consists of 52 items and is divided into six dimensions: health responsibility, self-actualization, physical activity, nutrition, interpersonal relationships, and stress management. The scale uses a 4-point Likert scale (1 representing “never” and 4 representing “always”), with a total score range of 52 to 208 points. A higher score indicates a higher level of health-promoting behavior. Based on the total score, behavioral levels were categorized into four grades: Poor (52–91 points), Fair (92–131 points), Good (132–171 points), and Excellent (172–208 points). Additionally, an item mean score of 2.5 was used as a cutoff; a score of ≥2.5 indicated that the individual was satisfied with their health-promoting behaviors. In this study, the overall Cronbach’s *α* coefficient for the scale was 0.920, with coefficients for each dimension ranging from 0.632 to 0.872, indicating good reliability.

#### 2.2.3 Chinese version of Heart Failure-Specific Health Literacy Scale (C-HFS-HLS)

The Heart Failure-Specific Health Literacy Scale was developed by Matsuoka et al. in Japan in 2016 to assess health literacy among patients with heart failure (27). The scale comprises 12 items across three dimensions: functional health literacy, interactive health literacy, and critical health literacy. Functional health literacy refers to the basic ability to read and understand health-related materials; interactive health literacy refers to the ability to obtain and apply health information using cognitive and communication skills; and critical health literacy refers to the ability to critically analyze health information and use it to support effective health management. The scale is rated on a 4-point Likert scale, with total scores ranging from 12 to 48. Items 1–4 are reverse-scored, whereas items 5–12 are positively scored; higher scores indicate higher levels of health literacy. Yue Meng et al. conducted cross-cultural adaptation of the scale and developed the Chinese version of the Heart Failure-Specific Health Literacy Scale (C-HFS-HLS), confirming its applicability among patients with heart failure in China (28). The Chinese version demonstrated good reliability and validity, with a Cronbach’s *α* coefficient of 0.900.

#### 2.2.4 Perceived Social Support Scale (MSPSS)

The Multidimensional Scale of Perceived Social Support (MSPSS) was developed by Zimet et al. (29). It is a self-report instrument designed to assess individuals’ perceived social support from multiple sources. The scale comprises 12 items. The original scale includes three dimensions: family support, friend support, and significant-other support. Jiang Qianjin adapted the scale to the Chinese cultural context by combining friend support and significant-other support into a single dimension, namely support outside the family, in which “significant others” referred to leaders, relatives, and colleagues (30). All items are rated on a 7-point Likert scale, and the total score reflects the level of perceived social support. Total scores are categorized into three levels: 12–36 indicates low support, 37–60 indicates moderate support, and 61–84 indicates high support. In the present study, the Cronbach’s *α* coefficient of the scale was 0.911.

#### 2.2.5 Consumer health activation index (CHAI)

The Consumer Health Activation Index was developed by Wolf et al. (31) and translated and adapted into Chinese by Zha Wang et al. (32) to assess health activation among patients with chronic diseases. The scale comprises three dimensions and 10 items. Each item is rated on a 6-point scale, ranging from 1 = “strongly disagree” to 6 = “strongly agree.” The raw score is converted linearly to a standardized score ranging from 0 to 100. Standardized scores of 0–79 indicate low health activation, 80–94 indicate moderate health activation, and 95–100 indicate high health activation. In the present study, the Cronbach’s *α* coefficient of the scale was 0.868.

### 2.3 Research hypotheses

Based on the COM-B model, this study constructed a determinant model of health-promoting behaviors among patients with chronic heart failure (CHF) across three dimensions: capability, opportunity, and motivation. Drawing on previous applications of the COM-B model and the disease characteristics of patients with CHF, this study defines health literacy as the capability factor, reflecting patients’ ability to access, understand, and apply health information; social support as the opportunity factor, representing external resources that provide emotional, informational, and practical assistance; and health activation as the motivation factor, reflecting patients’ intrinsic motivation to actively participate in health management and maintain healthy behaviors.

According to the COM-B model, behavioral change results from the joint effects of capability, opportunity, and motivation. Patients with higher health literacy and stronger social support are more likely to develop positive attitudes toward health management, thereby promoting the formation and maintenance of health-promoting behaviors. Previous studies have also shown that health literacy, social support, and health activation are closely and positively correlated, and that health activation may play an important mediating role in behavioral adaptation and behavioral change among patients with chronic diseases (33, 34). Based on the above theoretical analysis, this study proposes the following hypotheses:

*H1*: Health literacy has a direct positive effect on health-promoting behaviors among patients with CHF.

*H2*: Social support has a direct positive effect on health-promoting behaviors among patients with CHF.

*H3*: health activation has a direct positive effect on health-promoting behaviors among patients with CHF.

*H4*: health activation mediates the relationship between health literacy and health-promoting behaviors.

*H5*: Health activation mediates the relationship between social support and health-promoting behaviors.

### 2.4 Data collection

Participants were recruited strictly according to the predefined inclusion and exclusion criteria. Data were collected through on-site surveys. Participants who were able to read independently completed the questionnaire after receiving standardized instructions, whereas those with reading or literacy difficulties completed it with one-on-one assistance from trained researchers to ensure accurate understanding of the items. To minimize measurement error, data collection was conducted outside peak treatment hours in a quiet environment to reduce external disturbances. Questionnaires were checked immediately upon completion. On-site checks were performed to identify missing responses and duplicate submissions, thereby improving data completeness and authenticity. Before data entry, all questionnaires were reviewed for completeness, duplicate responses, and unreasonable answers, such as logical inconsistencies or outliers. Questionnaires with incomplete responses, obvious logical inconsistencies, or missing key variables were excluded. A total of 320 questionnaires were distributed and returned, yielding a response rate of 100.0%. During data entry and cleaning, 18 invalid questionnaires were excluded according to the quality-control criteria. The main reasons for exclusion were duplicate assessments (*n* = 4), incomplete questionnaires due to time constraints or emotional factors (*n* = 6), and missing key items (*n* = 8). Finally, 302 valid questionnaires from patients with CHF were included in the final analysis, yielding a valid questionnaire rate of 94.38%.

### 2.5 Data analysis

Statistical analyses were performed using SPSS version 26.0. Categorical variables are presented as frequencies and percentages, *n* (%). Normally distributed continuous variables are presented as mean ± standard deviation, whereas non-normally distributed variables are presented as median and interquartile range, M (P25, P75). Health-promoting behavior scores were used as the dependent variable, and sociodemographic and disease-related characteristics were used as independent variables to examine differences in health-promoting behaviors across participant subgroups. For normally distributed continuous variables, differences between two groups were analyzed using independent-samples t tests, whereas differences among multiple groups were analyzed using one-way analysis of variance when the assumptions of normality and homogeneity of variance were satisfied. If the assumptions of normality or homogeneity of variance were not satisfied, nonparametric tests, including the Mann–Whitney U test and Kruskal–Wallis H test, were used as appropriate. Spearman correlation analysis was used to examine associations among continuous variables. A two-sided *p* value < 0.05 was considered statistically significant.

Common method bias was assessed using Harman’s single-factor test. Multiple linear regression analysis was performed using health-promoting behavior scores as the dependent variable. Variables that were statistically significant in univariate or correlation analyses (*p* < 0.05) were entered into the regression model as independent variables using the enter method. Before interpreting the multiple linear regression model, regression assumptions were examined. Multicollinearity was assessed using the variance inflation factor (VIF) and tolerance values. Residual diagnostics were performed to evaluate the normality and homoscedasticity of residuals. Specifically, the histogram and normal probability plot of standardized residuals were inspected to assess residual normality, and the scatterplot of standardized residuals against standardized predicted values was examined to assess homoscedasticity.

Based on the COM-B model, an observed-variable path analysis model was constructed using AMOS version 23.0 to examine the associations among health literacy, perceived social support, health activation, and health-promoting behaviors. The variables in the model were represented by validated scale scores or dimension scores rather than by item-level latent measurement models. Model parameters were estimated using maximum likelihood estimation. Model fit was evaluated using χ^2^/df, GFI, AGFI, IFI, TLI, CFI, and RMSEA. Mediating effects were tested using the bias-corrected bootstrap method with 5,000 resamples and 95% confidence intervals. Because the purpose of this study was to examine theoretically informed pathways among established constructs rather than to develop or revalidate measurement instruments, the findings should be interpreted as observed-variable path associations rather than as evidence from a full item-level latent-variable model.

Nonlinear analysis was performed using Python version 3.9. The target variable was the total health-promoting behavior score, and sociodemographic, disease-related, health literacy, perceived social support, and health activation variables were included as candidate predictors. Categorical variables were numerically encoded according to the predefined coding strategy used in the regression analysis, and continuous variables were retained as numerical variables.

The dataset was randomly divided into a training set and a test set at a ratio of 7:3, with a fixed random seed of 42 for data splitting and model training. StandardScaler was fitted on the training set and then applied to the test set to reduce the influence of different measurement scales and avoid data leakage. A random forest regression model was constructed using RandomForestRegressor. Hyperparameter optimization was conducted using Optuna with 50 trials, and 3-fold cross-validation was used within the training set. The tuned hyperparameters included n_estimators ranging from 100 to 1,000, max_depth ranging from 3 to 20, min_samples_split ranging from 2 to 20, min_samples_leaf ranging from 1 to 10, and max_features selected from “sqrt” and “log2.” The optimization objective was to maximize the negative mean squared error. The final model was trained using the optimal hyperparameter combination selected by Optuna. Model performance was evaluated in both the training and test sets using mean squared error, root mean squared error, mean absolute error, and the coefficient of determination. SHAP values were calculated using TreeExplainer to interpret the contribution of each feature to model predictions. SHAP summary plots, global feature importance plots, dependence plots, heatmaps, force plots, and interaction plots were generated to visualize feature importance and potential nonlinear associations. The summary and global importance plots were used to visualize the overall contribution and relative importance of the features. The random forest analysis was used as a supplementary exploratory analysis to examine nonlinear associations and feature importance, rather than as a confirmatory hypothesis-testing method. To reduce the risk of overfitting and improve model stability, the dataset was divided into training and test sets, and cross-validation with hyperparameter optimization was performed. Therefore, the machine-learning results were interpreted as complementary evidence and should be verified in larger independent samples.

### 2.6 Ethical consideration

This study was approved by the Ethics Review Committee of Shanghai Municipal Hospital of Traditional Chinese Medicine, Shanghai University of Traditional Chinese Medicine (Approval No. 2025SHL-KY-06-02), and complied with the ethical requirements for medical research. The study was conducted in accordance with the Declaration of Helsinki and relevant ethical guidelines to ensure full protection of participants’ rights and interests. Before the study began, the researchers explained the study purpose, procedures, and methods to all participants and fully informed them of potential risks and expected benefits, ensuring that participation was based on adequate understanding and voluntary informed consent. Written informed consent was obtained from all participants. Participants were informed that their participation was entirely voluntary and that they could withdraw from the study at any stage without any effect on their subsequent medical care. Participants’ privacy and personal information were strictly protected throughout the study. All questionnaires and data were recorded anonymously and used solely for academic research purposes.

## 3 Result

### 3.1 Participants’ basic information

A total of 320 questionnaires were distributed, and 302 valid questionnaires were included in the analysis, yielding a valid response rate of 94.38%. Among the 302 patients with CHF, 17 patients (5.63%) were aged <50 years, 156 (51.66%) were aged 50–74 years, and 129 (42.72%) were aged ≥75 years. Other sociodemographic and disease-related characteristics are presented in Table 1. Health-promoting behavior scores differed significantly across age group, educational level, employment status, marital status, living arrangement, monthly household income, type of medical insurance, disease duration, comorbidity status, and number of CHF-related hospitalizations in the past year (*p* < 0.05). Detailed results are shown in Table 1.

**Table 1:** Univariate analysis of health promotion behaviors in CHF patients versus general demographic data (*n* = 302).

Variables	Category	*n*	Percentage (%)	*M*(*P*_25_, *P*_75_)	Test statistic	*p* value
Gender	Male	178	58.94	124.00 (117.00, 136.00)	−1.108*	0.268
	Female	124	41.06	129.00 (115.00, 141.00)		
Age	< 50	17	5.63	133.00 (133.00, 137.00)	10.315	0.006
	50 ~ 74	156	51.66	128.50 (114.75, 146.50)		
	≥ 75	129	42.72	124.00 (118.00,134.00)		
BMI	< 24	136	45.03	127.00 (118.00, 143.25)	5.695	0.058
	24 ~ 27.9	131	43.38	125.00 (114.00, 135.50)		
	≥ 28	35	11.59	124.00 (116.50, 132.00)		
Education	Junior high school or below	62	20.53	118.50 (105.50, 126.00)	36.966	< 0.001
	Senior high school or technical secondary school	103	34.11	124.00 (117.00, 136.50)		
	Junior college, bachelor’s degree, or above	137	45.36	133.00 (121.00, 151.00)		
Employment status	Employed	41	13.58	150.00 (134.00, 174.00)	−7.133*	< 0.001
	Retired	261	86.42	124.00 (115.00, 134.00)		
Marital status	Unmarried/divorced/widowed	78	25.83	114.00 (103.50, 128.00)	−6.580*	< 0.001
	Married	224	74.17	130.00 (120.25, 139.00)		
Living arrangement	Living alone	43	14.24	113.00 (90.50, 139.00)	−6.025*	< 0.001
	Living with spouse or children	259	85.76	128.00 (119.00, 139.00)		
Monthly household income	< 3,000	21	6.95	89.00 (86.00, 114.00)	73.185	< 0.001
	3,000 ~ 5,000	83	27.48	121.00 (115.50, 125.00)		
	> 5,000	198	65.56	132.50 (119.25, 145.75)		
Type of medical insurance	Employee medical insurance	228	75.50	129.00 (118.00, 143.25)	28.501	< 0.001
	Resident medical insurance	65	21.52	122.00 (117.00, 130.00)		
	Out-of-pocket payment	9	2.98	112.00 (89.00, 113.00)		
Disease duration	< 2	144	47.68	130.00 (118.75, 138.00)	8.264	0.016
	2 ~ 5	89	29.47	128.00 (115.00, 137.00)		
	> 5	69	22.85	119.00 (113.00, 130.00)		
Comorbidity status	0	26	8.61	135.50 (133.00, 144.00)	65.855	< 0.001
	1	161	53.31	129.00 (121.00, 145.00)		
	2 ~ 3	47	15.56	125.00 (115.50, 137.00)		
	≥4	68	22.52	116.00 (107.75, 119.00)		
NYHA functional class	II	179	59.27	126.00 (113.00, 137.00)	3.339	0.188
	III	100	33.11	126.50 (118.00, 137.00)		
	IV	23	7.62	123.00 (118.00, 127.00)		
Number of hospitalizations in the past year	0	109	36.09	139.00 (134.00, 155.00)	130.044	< 0.001
	1 ~ 3	153	50.66	118.00 (113.00, 128.00)		
	> 3	40	13.25	120.00 (112.75, 122.25)		

*Indicates the Mann–Whitney U test; all other results are from the Kruskal-Wallis H test.

### 3.2 Scores of health literacy, perceived social support, and health activation among patients with CHF

Among the 302 patients with CHF, health-promoting behavior scores were not normally distributed. The total score was 126.00 (116.00, 137.00), and the mean item score was 2.42 (2.23, 2.63), indicating an overall moderate level. Scores differed across dimensions, with higher scores observed for self-actualization and nutrition and lower scores for health responsibility and physical activity; physical activity had the lowest score among all dimensions (Table 2). According to the classification of health-promoting behaviors, most patients were categorized as having moderate or good levels, whereas relatively few were classified as having poor or excellent levels (Table 3). The total heart failure–specific health literacy score was 26.00 (23.00, 28.00). Among its dimensions, functional health literacy scored highest, followed by interactive health literacy, whereas critical health literacy scored lowest. The total perceived social support score was 63.00 (53.00, 72.00). Family support scored highest, followed by significant-other support, whereas friend support scored lowest. The raw health activation score was 39.50 (32.25, 45.00), and the converted standardized score was 59.00 (44.50, 70.00).

**Table 2:** Health promotion behaviors and their dimensional scores in CHF patients (*n* = 302).

Variables	Items	Possible range	Observed range	Median (P25, P75)	Item score, Median (P25, P75)	Rank
Self-actualization	9	9 ~ 36	12 ~ 36	29.50 (21.00, 32.00)	3.28 (2.33, 3.56)	1
Nutrition	9	9 ~ 36	12 ~ 34	25.00 (20.00, 30.00)	2.78 (2.22, 3.33)	2
Stress management	8	8 ~ 32	14 ~ 30	21.00 (17.00, 24.00)	2.63 (2.13, 3.00)	3
Interpersonal relations	9	9 ~ 36	9 ~ 36	21.00 (18.00, 25.00)	2.33 (2.00, 2.78)	4
Health responsibility	9	9 ~ 36	9 ~ 34	20.00 (16.00, 23.00)	2.22 (1.78, 2.56)	5
Physical activity	8	8 ~ 32	8 ~ 30	15.00 (11.00, 17.00)	1.88 (1.38, 2.13)	6
Total score	52	52 ~ 208	78 ~ 200	126.00 (116.00, 137.00)	2.42 (2.23, 2.63)	

**Table 3:** Levels of health-promoting behaviors among CHF patients (*n* = 302).

Rank	Possible range	*n*	Percentage (%)	Minimum	Maximum	Total score, Median (P25, P75)
Poor	52 ~ 90	14	4.64	78	90	85.50 (84.00, 88.00)
Moderate	91 ~ 129	158	52.32	91	129	118.00 (113.00, 124.00)
Good	130 ~ 168	104	34.44	130	168	137.00 (133.00, 145.00)
Excellent	169 ~ 208	26	8.61	170	200	182.00 (173.00, 178.00)

Among the dimensions of health activation, self-efficacy had the highest score, followed by knowledge and action. Spearman correlation analysis showed that health literacy, perceived social support, and health activation were positively correlated with the total health-promoting behavior score among patients with CHF (*p* < 0.01), with correlation coefficients of 0.394, 0.362, and 0.382, respectively (Tables 4, 5).

**Table 4:** Correlation analysis between CHF health promotion behaviors and various variables.

Independent variable	Health literacy	Social support	Health activation	Health-promoting behaviors
Health literacy	1			
Social support	0.434**	1		
Health activation	0.451**	0.453**	1	
Health-promoting behaviors	0.394**	0.362**	0.382**	1

**Indicates *p* < 0.01; * indicates *p* < 0.05.

**Table 5:** Common method bias test results.

No	Eigenvalue	Percentage of variance	Cumulative variance explained (%)
1	18.763	21.322	21.322
2	4.449	5.056	26.378
3	4.082	4.639	31.017
4	4.002	4.548	35.565
5	2.999	3.407	38.972
6	2.874	3.266	42.238
7	2.557	2.906	45.144
8	2.310	2.625	47.769
9	2.108	2.395	50.164
10	1.991	2.262	52.426
11	1.928	2.191	54.617
12	1.797	2.042	56.659
13	1.763	2.004	58.663
14	1.611	1.831	60.494
15	1.329	1.511	62.005
16	1.211	1.376	63.381
17	1.181	1.342	64.723
18	1.113	1.265	65.988
19	1.056	1.200	67.188
20	1.035	1.176	68.365
21	1.018	1.157	69.522
22	0.988	1.123	70.644
23	0.969	1.101	71.746

### 3.3 Multiple linear regression analysis of health promotion behaviors in CHF patients

Harman’s single-factor test was used as a preliminary assessment of common method bias. The analysis identified 20 factors with eigenvalues greater than 1, and the first unrotated factor accounted for 21.32% of the total variance, which was below the commonly used threshold of 40%. The detailed distribution of these proportions is presented in Table 5. This result suggested that common method bias was unlikely to be severe. However, Harman’s single-factor test cannot definitively rule out common method variance. This study used health-promoting behaviors among patients with CHF as the dependent variable. Health literacy, perceived social support, health activation, and demographic and clinical characteristics that were statistically significant in the univariate or correlation analyses were entered as independent variables. The variable assignment method is shown in Table 6. Multiple linear regression analysis was performed using the enter method, and the results are shown in Table 7.

**Table 6:** Independent variable assignment method.

Independent variable	Assignment
Age	Reference: <50 years; 50–74 years (X1 = 1, X2 = 0); ≥75 years (X1 = 0, X2 = 1)
Educational level	Reference: junior high school or below; senior high school/technical secondary school (X1 = 1, X2 = 0); junior college or above (X1 = 0, X2 = 1)
Employment status	Employed = 1; retired = 2
Marital status	Unmarried/divorced/widowed = 1; married = 2
Living arrangement	Living alone = 1; living with spouse/children = 2
Monthly household income	Reference: < 3,000; 3,000–5,000 (X1 = 1, X2 = 0); > 5,000 (X1 = 0, X2 = 1)
Type of medical insurance	Reference: employee medical insurance; resident medical insurance (X1 = 1, X2 = 0); out-of-pocket payment (X1 = 0, X2 = 1)
Disease duration	Reference: <2 years; 2–5 years (X1 = 1, X2 = 0); >5 years (X1 = 0, X2 = 1)
Comorbidity status	Reference: no comorbidities; one comorbidity (X1 = 1, X2 = 0, X3 = 0); two to three comorbidities (X1 = 0, X2 = 1, X3 = 0); four or more comorbidities (X1 = 0, X2 = 0, X3 = 1)
Number of hospitalizations in the past year	Reference: 0 hospitalizations; 1–3 hospitalizations (X1 = 1, X2 = 0); >3 hospitalizations (X1 = 0, X2 = 1)
Health literacy	Entered as the original value
Social support	Entered as the original value
Health activation	Entered as the original value

**Table 7:** Multiple linear regression analysis of health promotion behaviors in CHF patients (*n* = 302).

Variable	*B*	*SE*	*β*	*t*	*p*	VIF	Tolerance
Constant	97.244	8.415		11.556	<0.001		
Senior high school/technical secondary school, educational level: reference = junior high school or below	0.668	2.315	0.014	0.289	0.773	2.367	0.423
Junior college or above, educational level: reference = junior high school or below	1.006	2.560	0.023	0.393	0.694	3.192	0.313
Age 50 ~ 74 years, reference = <50 years	−2.096	3.296	−0.047	−0.636	0.525	5.334	0.187
Age ≥75 years, reference = <50 years	−3.938	3.403	−0.088	−1.157	0.248	5.570	0.180
Monthly household income 3,000–5,000 yuan, reference = <3,000 yuan	7.927	3.868	0.160	2.050	0.041	5.861	0.171
Monthly household income >5,000 yuan, reference = <3,000 yuan	11.462	3.792	0.246	3.022	0.003	6.384	0.157
Resident medical insurance, reference = employee medical insurance	−7.014	2.067	−0.130	−3.393	0.001	1.419	0.705
Out-of-pocket payment, reference = employee medical insurance	−11.835	4.687	−0.091	−2.525	0.012	1.249	0.801
Disease duration 2–5 years, reference = <2 years	3.082	1.820	0.064	1.693	0.092	1.353	0.739
Disease duration >5 years, reference = <2 years	0.631	1.976	0.012	0.319	0.750	1.354	0.739
One comorbidity, reference = no comorbidities	−1.332	2.910	−0.030	−0.458	0.648	4.144	0.241
Two to three comorbidities, reference = no comorbidities	−3.668	3.354	−0.060	−1.094	0.275	2.906	0.344
Four or more comorbidities, reference = no comorbidities	−13.378	3.195	−0.253	−4.187	< 0.001	3.502	0.286
1–3 hospitalizations in the past year, reference = 0 hospitalizations	−11.274	1.864	−0.255	−6.049	< 0.001	1.707	0.586
>3 hospitalizations in the past year, reference = 0 hospitalizations	−12.915	2.665	−0.198	−4.847	< 0.001	1.604	0.623
Retired, reference = employed	−9.297	2.686	−0.144	−3.461	0.001	1.664	0.601
Married, reference = unmarried/divorced/widowed	−0.767	2.416	−0.015	−0.317	0.751	2.198	0.455
Living with spouse/children, reference = living alone	5.42	3.130	0.086	1.731	0.084	2.353	0.425
Health activation	0.238	0.041	0.224	5.732	< 0.001	1.470	0.680
Social support	0.263	0.066	0.163	3.998	< 0.001	1.590	0.629
Health literacy	0.611	0.167	0.139	3.654	< 0.001	1.393	0.718

*F* = 32.494, *p* < 0.001, *R* = 0.842, *R*^2^ = 0.709, adjusted *R*^2^ = 0.687.

Regression diagnostic analyses were performed before interpreting the final model. The standardized residuals ranged from −2.650 to 2.910, with no values exceeding ±3, suggesting that no extreme residual outliers were observed. The histogram of standardized residuals showed an approximately bell-shaped distribution, and the normal probability plot showed that the observed cumulative probabilities were close to the expected cumulative probabilities, indicating that the residuals were approximately normally distributed. The scatterplot of standardized residuals against standardized predicted values showed no obvious funnel-shaped pattern, suggesting that the assumption of homoscedasticity was generally acceptable. Multicollinearity diagnostics showed that the VIF values of all predictors ranged from 1.249 to 6.384, and the corresponding tolerance values were all above 0.10. These results suggested that severe multicollinearity was unlikely. However, several demographic dummy variables had VIF values greater than 5, indicating the possibility of moderate multicollinearity among some covariates. Therefore, the regression coefficients for these demographic variables should be interpreted with caution. For the three key COM-B-related psychological variables, the VIF values were 1.393 for health literacy, 1.590 for perceived social support, and 1.470 for health activation, with corresponding tolerance values of 0.718, 0.629, and 0.680, respectively, indicating low multicollinearity among the core psychological predictors. In addition, the correlation matrix showed moderate correlations among health literacy, perceived social support, and health activation, with coefficients ranging from 0.434 to 0.453, suggesting that these constructs were conceptually related but not statistically redundant.

After controlling for other potential confounders, monthly household income, type of medical insurance, number of comorbidities, number of hospitalizations in the past year, employment status, health activation, perceived social support, and health literacy were independently associated with health-promoting behaviors among patients with CHF (*p* < 0.05). Higher monthly household income, greater health activation, higher perceived social support, and higher health literacy were positively associated with better health-promoting behaviors. In contrast, resident medical insurance or out-of-pocket payment compared with employee medical insurance, having four or more comorbidities, having at least one hospitalization in the past year, and being retired were negatively associated with health-promoting behaviors.

### 3.4 Path analysis of factors associated with health-promoting behaviors in patients with CHF

**Figure 1:**
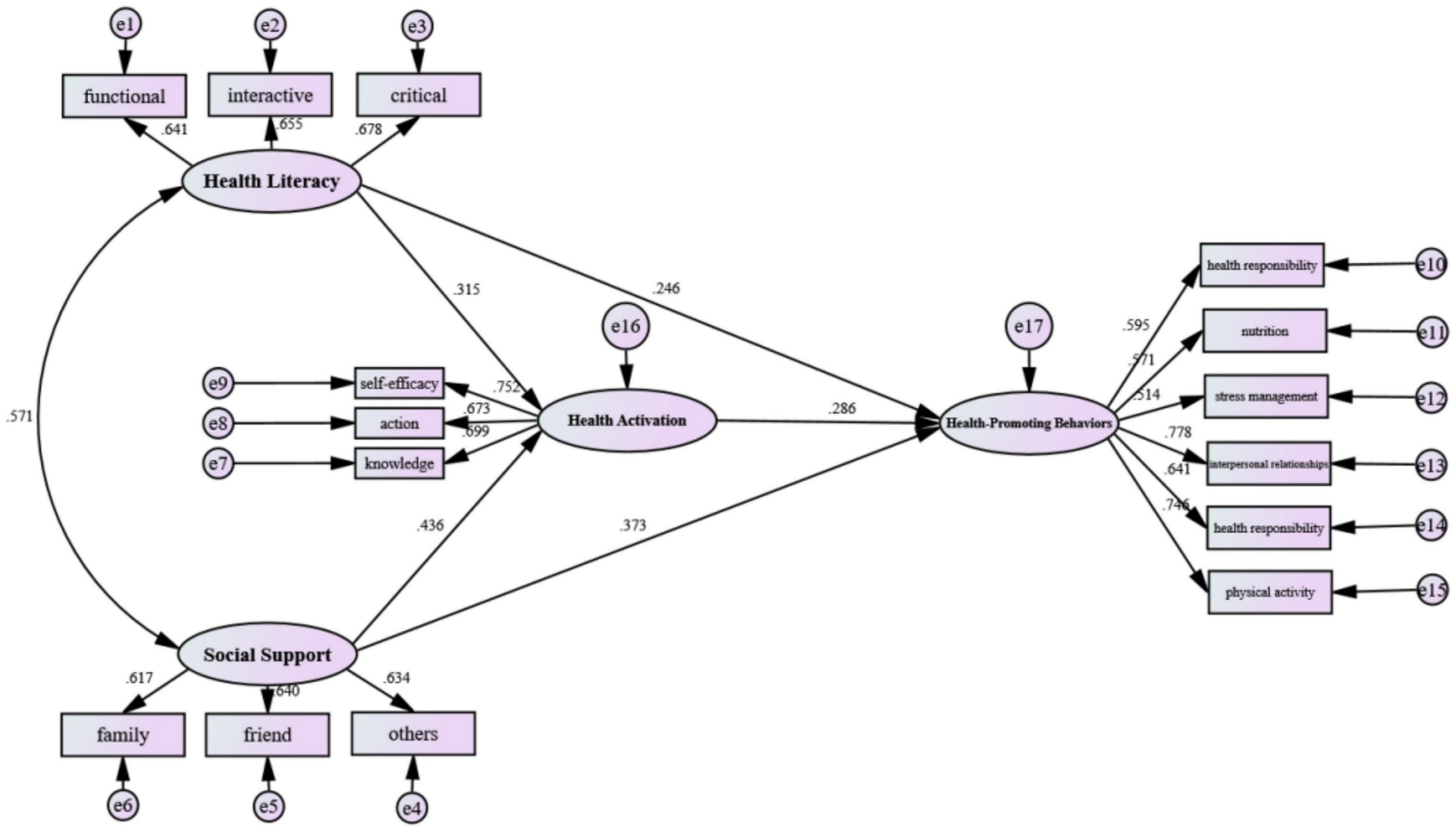
Path model of health literacy, health activation, and health-promoting behaviors.

This study employed the COM-B model as its theoretical foundation and developed a path analysis model of factors associated with health-promoting behaviors in patients with CHF based on prior research hypotheses. The model further examined the mediating pathways among health literacy, perceived social support, health activation, and health-promoting behaviors. The path analysis model was constructed with health literacy and perceived social support as independent variables, health activation as the mediator, and health-promoting behaviors as the dependent variable (Figure 1). Results demonstrated satisfactory model fit, confirming good model adequacy without requiring further modifications (Table 8). Hypothesis testing of the path analysis model revealed that all path coefficients exhibited statistically significant effects (Table 9).

**Table 8:** Results of the goodness-of-fit test for the path analysis model.

Fit index	χ2/df	GFI	AGFI	IFI	TLI	CFI	RMSEA
Observed value	1.470	0.951	0.930	0.970	0.962	0.970	0.040
Recommended criterion	< 3	> 0.9	> 0.9	> 0.9	> 0.9	> 0.9	< 0.08

**Table 9:** Path coefficients of the path analysis model for health-promoting behaviors in patients with CHF (*n* = 302).

Path relationship	Standardized path coefficient	*SE.*	*CR.*	*p*
Health literacy → Health activation	0.315	0.190	3.062	0.002
Social support → Health activation	0.436	0.055	3.967	< 0.001
Health activation → Health-promoting behaviors	0.286	0.136	2.995	0.003
Health literacy → Health-promoting behaviors	0.246	0.244	2.660	0.008
Social support → Health-promoting behaviors	0.373	0.076	3.453	< 0.001

### 3.5 Mediation effect analysis of the path analysis model for health-promoting behaviors in patients with CHF

Based on the path analysis results, the hypothesized pathways were supported. To further examine the mediating effects, the bias-corrected bootstrap method was performed with 5,000 resamples and 95% confidence intervals. The results showed that health literacy and social support had significant indirect associations with health-promoting behaviors through health activation, with indirect effect values of 0.090 and 0.125, respectively (*p* = 0.017 and *p* = 0.006). These findings suggest that health literacy and social support were positively and indirectly associated with health-promoting behaviors through health activation. The total effects of health literacy and social support on health-promoting behaviors were 0.336 and 0.497, respectively. The mediating effects accounted for 26.78 and 25.15% of the total effects, respectively, indicating that health activation played a partial mediating role in these associations. Detailed results are presented in Table 10.

**Table 10:** Analysis of mediating effects among variables in the model.

Path	Effect type	Effect size	Standard error	95% confidence interval		*p*
				Lower limit	Upper limit	
Health literacy → Health activation → Health-promoting behaviors	Total effect	0.336	0.095	0.119	0.506	0.006
	Direct effect	0.246	0.101	0.015	0.419	0.041
	Indirect effect	0.090	0.054	0.013	0.239	0.017
Social support → Health activation → Health-promoting behaviors	Total effect	0.497	0.085	0.335	0.679	0.000
	Direct effect	0.373	0.099	0.200	0.591	0.002
	Indirect effect	0.125	0.054	0.045	0.270	0.006

### 3.6 Nonlinear importance ranking of factors influencing health promotion scores in CHF patients

**Figure 2:**
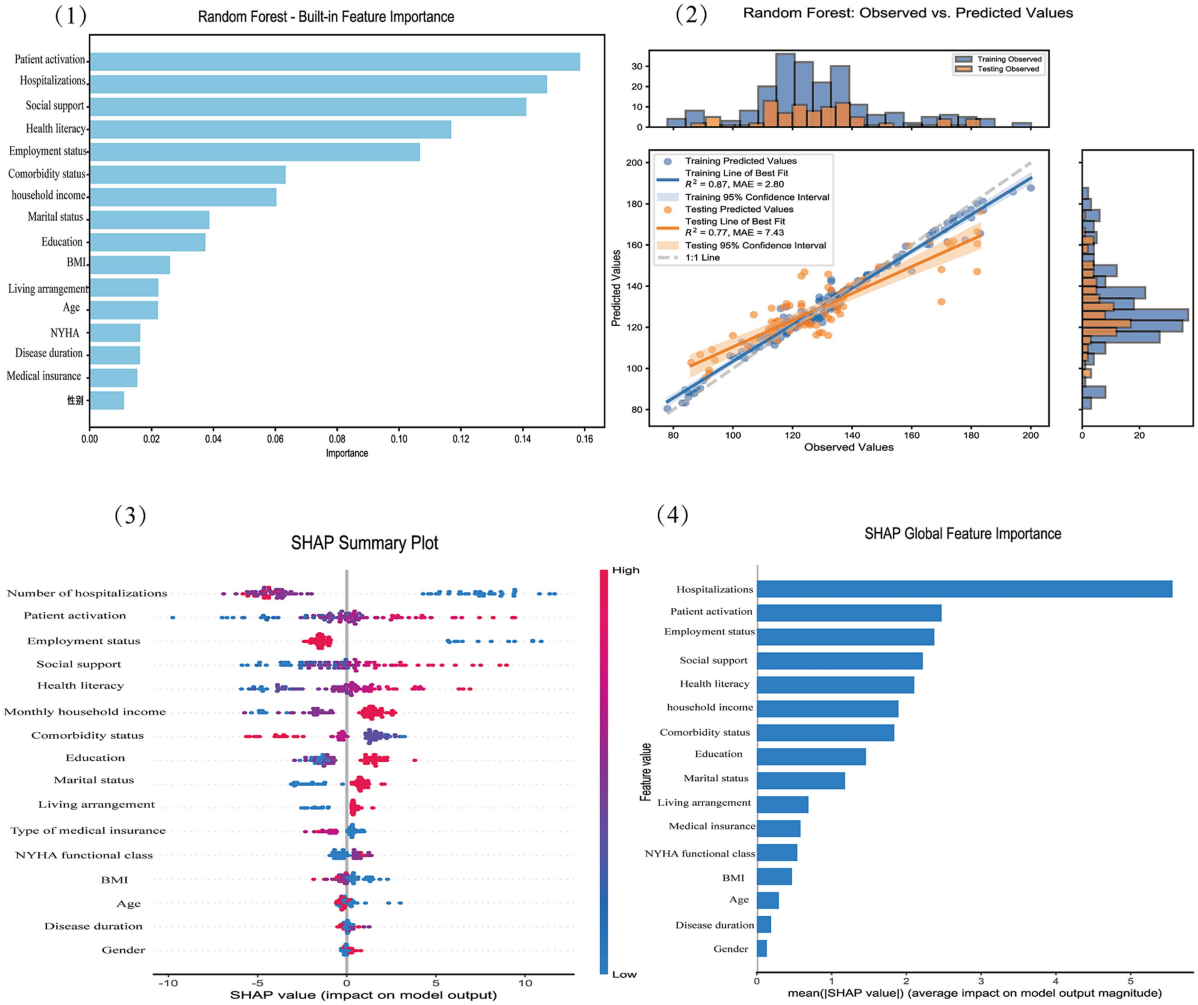
Nonlinear importance ranking and model interpretation of factors influencing health-promoting behavior scores in patients with chronic heart failure. (1) Feature importance ranking derived from the random forest regression model. (2) Predictive performance of the random forest model in the training and test sets. (3) SHAP summary plot showing the direction and magnitude of each predictor’s contribution to individual predictions; each point represents one patient, and the color gradient indicates the relative feature value. (4) SHAP-based global feature importance ranking based on mean absolute SHA*p* values. Overall, number of hospitalizations in the past year, health activation, total perceived social support score, and total health literacy score were the main contributors to the prediction of health-promoting behavior scores.

According to the feature importance ranking of the random forest model, the most important predictors were health activation, number of hospitalizations, total perceived social support score, total health literacy score, employment status, comorbidity status, income level, marital status, educational level, BMI, living arrangement, age, NYHA functional class, disease duration, type of medical insurance, and sex. Details are shown in Figure 2 (1). As shown in Figure 2 (2), the model showed good predictive performance in both the training and test sets. In the training set, the R^2^ was 0.87 and the MAE was 2.80, indicating good model fit and relatively low prediction error. In the test set, the R^2^ was 0.77, suggesting acceptable generalization performance. Overall, the model demonstrated acceptable predictive performance, with no marked decline from the training set to the test set.

The SHAP summary plot showed that different features contributed differently to the prediction of health-promoting behaviors [Figure 2 (3)]. Each point represents the SHAP value of an individual sample, and the color gradient from blue to red indicates increasing feature values. Larger absolute SHAP values indicate greater contributions to the model output. The results indicated that number of hospitalizations, health activation, total health literacy score, and total perceived social support score were among the most influential predictors. The SHAP values for number of hospitalizations showed a wide distribution, indicating a strong contribution to model predictions. In addition, higher health activation and higher total health literacy scores were associated with higher predicted health-promoting behavior scores. In contrast, educational level, marital status, and sex showed more concentrated SHAP value distributions, suggesting relatively smaller contributions to the prediction. BMI, employment status, comorbidity status, living arrangement, and type of medical insurance also contributed to the model, but their contributions were smaller than those of number of hospitalizations and health literacy.

The SHAP global feature importance ranking showed that the most important variables were number of hospitalizations in the past year, health activation, employment status, total perceived social support score, total health literacy score, monthly per capita household income, comorbidity status, educational level, marital status, living arrangement, type of medical insurance, NYHA functional class, BMI, age, disease duration, and sex [Figure 2 (4)]. Comparison of SHAP-based global feature importance with the built-in feature importance of the random forest model showed that the two methods generally identified similar key predictors. In particular, number of hospitalizations in the past year, health activation, total perceived social support score, and total health literacy score ranked highly in both methods, suggesting that these variables contributed substantially to the prediction of health-promoting behaviors. However, some differences were observed in the relative rankings. For example, employment status and monthly per capita household income ranked higher in the SHAP analysis, whereas BMI, age, disease duration, and sex ranked lower in both methods. These differences may be explained by the different evaluation mechanisms of the two methods. The built-in feature importance of the random forest model mainly reflects each feature’s contribution to node splitting, whereas SHAP values quantify the marginal contribution of each feature to individual predictions, thereby providing both global and local interpretability.

## 4 Discussion

### 4.1 The health-promoting behaviors of CHF patients have not reached an ideal level

The results showed that the overall level of health-promoting behaviors among patients with CHF was moderate, suggesting that most patients had not yet established stable and optimal health behavior patterns in daily disease management. This finding is consistent with those of Habibzadeh et al. and Liu et al. (35, 36). Moreover, the score was lower than the reported level of health-promoting lifestyles in the general population (37). This suggests that although patients with CHF may have some awareness of health behaviors, they still face practical barriers to the consistent and systematic implementation of health-promoting behaviors. Across dimensions, the scores ranked from highest to lowest as follows: self-actualization, nutrition, stress management, interpersonal relations, health responsibility, and physical activity. Physical activity had the lowest score, which is consistent with previous studies (35, 36), suggesting that physical activity remains a weak component of health-promoting behaviors among patients with CHF. Patients with CHF often experience impaired cardiac function, reduced exercise tolerance, and discomfort after physical activity. Together with concerns about exercise-related risks, these symptoms may lead to avoidance of physical activity and reduced adherence to regular exercise. In addition, insufficient professional exercise guidance and limited self-monitoring ability may further restrict engagement in physical activity. Classification results showed that most patients with CHF were at the moderate or good level, whereas only a small proportion reached the excellent level. This finding is consistent with Tierney et al. (38) and further indicates that health-promoting behaviors among patients with CHF remain suboptimal. These findings suggest that conventional health education alone may be insufficient to achieve sustained improvements in health-promoting behaviors. Health-promoting behaviors are complex and may be influenced by multiple factors, including physical condition, psychological status, and the social environment. Therefore, systematic, stratified, and stepwise intervention strategies are needed.

### 4.2 Analysis of influencing factors on health promotion behaviors in CHF patients

The results of the linear and nonlinear analyses showed that several sociodemographic and clinical characteristics were associated with health-promoting behaviors among patients with CHF, suggesting that these behaviors are shaped not only by individual behavioral mechanisms but also by social context and disease burden. The number of hospitalizations in the past year was negatively associated with health-promoting behaviors and ranked highly in the nonlinear analysis, indicating its potential predictive value. Frequent hospitalizations may reflect poor disease control, disease progression, or multiple comorbidities, which can limit physical function and behavioral capacity and reduce patients’ confidence in engaging in health-promoting behaviors. Repeated hospitalizations may also intensify feelings of helplessness, disease-related fear, and loss of control, thereby reducing patients’ initiative to adopt and maintain health-promoting behaviors (39). These findings suggest that clinical care should pay greater attention to patients with repeated hospitalizations by strengthening symptom monitoring, post-discharge follow-up, psychological support, and individualized behavioral guidance to improve their participation in and adherence to health-promoting behaviors.

In this study, household income was positively associated with health-promoting behaviors among patients with CHF (40). Compared with patients with lower income, those with higher income showed better health-promoting behaviors, which may be related to greater access to medical resources, nutritional support, rehabilitation services, and health information (41). Having four or more comorbidities was negatively associated with health-promoting behaviors among patients with CHF. Patients with multiple comorbidities had lower health-promoting behavior scores than those with no or fewer comorbidities, which is consistent with the findings of Wister et al. (42) on the association between multimorbidity and health-promoting behaviors in older adults. Patients with multimorbidity need to manage multiple conditions simultaneously, which may increase treatment burden, complicate behavior implementation, and obscure priorities in health management, thereby limiting the sustained practice of health-promoting behaviors.

In this study, health literacy, perceived social support, and health activation were significantly associated with health-promoting behaviors and ranked highly in both linear and nonlinear analyses. Within the COM-B framework, these three constructs correspond to capability, opportunity, and motivation, respectively. Health literacy reflects patients’ ability to access, understand, evaluate, and apply health information in disease management (43). Patients with CHF need to manage medication use, symptom monitoring, diet, physical activity, and self-care on a long-term basis (44). Limited health literacy may reduce their ability to translate disease-related information into daily health-promoting behaviors. This finding is consistent with previous studies showing that health literacy is closely related to health-promoting behaviors and self-management among patients with chronic diseases (45, 46). The relatively low score for critical health literacy also indicates that some patients may have difficulty evaluating and applying health information, highlighting the need for tiered education and skills-based guidance.

Perceived social support was also positively associated with health-promoting behaviors, which is consistent with previous research (47, 48). Social support provides emotional, informational, and practical resources that create opportunities for behavior change. Support from family members may facilitate medication adherence, dietary control, and physical activity, whereas support from healthcare professionals and peers may reduce uncertainty and improve behavioral confidence. This finding is also consistent with Xin et al. (49), who emphasized the role of social support in reducing self-regulation fatigue and strengthening behavioral confidence. Given that many patients with CHF are middle-aged or older adults and rely heavily on family-based care, interventions should strengthen family involvement, peer support, and effective clinician–patient communication.

Health activation was another important factor associated with health-promoting behaviors and showed high predictive importance in the nonlinear analysis. As a motivational component in the COM-B framework, health activation reflects patients’ readiness and willingness to seek health information, participate in health-related decision-making, and take action (50). Patients with higher health activation may be more likely to engage actively in disease management and maintain behaviors such as symptom monitoring, dietary regulation, and appropriate physical activity. This result is consistent with Greene et al. (51). Ho et al. (52) also emphasized the important role of patients’ subjective initiative in health behavior change. Therefore, interventions should not focus solely on knowledge delivery or external support. Motivational strategies, including motivational interviewing, shared goal setting, role modeling, and positive feedback, may help enhance health activation and support sustained behavioral change.

The interpretation of these findings should also consider the conceptual overlap among health literacy, perceived social support, and health activation. These constructs are theoretically related within the COM-B framework, and moderate associations among them are expected. In this study, the correlation matrix and multicollinearity diagnostics suggested no serious redundancy among the core psychological predictors. However, several demographic dummy variables showed possible moderate multicollinearity; therefore, the regression coefficients for these covariates should be interpreted with caution. Future studies may further examine the distinct and shared contributions of capability, opportunity, and motivation using longitudinal designs and larger samples.

### 4.3 Path analysis of factors associated with health-promoting behaviors among patients with CHF

Path analysis showed that health literacy and perceived social support were directly and indirectly associated with health-promoting behaviors through health activation. Because this was a cross-sectional study, these mediation pathways should be interpreted as statistical associations consistent with the COM-B framework rather than as causal mechanisms. Within the COM-B framework, health literacy represents capability, perceived social support represents opportunity, and health activation represents motivation. Higher health literacy may help patients better understand the value of health-promoting behaviors, whereas stronger perceived social support may provide emotional, informational, and practical resources for behavior implementation. These factors may be linked to higher health activation, which reflects patients’ willingness to participate in health management and translate available knowledge and support into action. Previous evidence has also shown that patients with higher health activation are more likely to engage actively in disease management and adopt preventive health behaviors (51). This pathway is consistent with the capability–opportunity–motivation–behavior logic of the COM-B framework and previous evidence on motivation in health behavior change (53).

Clinically, health education and social support alone may be insufficient if patients lack the motivation and confidence to act. Enhancing health activation may therefore be an important entry point for improving health-promoting behaviors among patients with CHF. Strategies such as stratified education, family involvement, peer support, motivational interviewing, goal setting, and positive feedback may help patients move from passive receipt of care to active participation in long-term disease management.

### 4.4 Clinical implications

The results of this study showed that the overall level of health-promoting behaviors among patients with CHF requires improvement and that the development and maintenance of these behaviors are associated with multiple factors, including health literacy, social support, and health activation. Based on the COM-B model and the present findings, several implications can be proposed for interventions targeting health-promoting behaviors among patients with CHF. First, future clinical interventions should focus on improving health literacy as the foundation for strengthening patients’ capability to engage in health-promoting behaviors. Health education should move beyond the traditional model of information dissemination. Instead, education should be understandable, progressive, and tailored to patients’ cognitive levels and comprehension abilities. The focus should be on improving patients’ understanding and application of disease knowledge, symptom recognition, physical activity safety, and self-management skills. Scenario-based practice, practical demonstrations, and feedback-based guidance may help patients translate health knowledge into action and improve their ability to implement health-promoting behaviors. Second, the social support environment should be optimized to expand opportunities for implementing health-promoting behaviors. The findings suggest that social support plays an important role in facilitating health-promoting behaviors. Nursing interventions should emphasize family involvement. Family health education and caregiving guidance may improve the quality of family support and enable family members to play an active role in patients’ daily health management. In addition, access to social support may be expanded through peer support groups, online follow-up, and remote guidance. These strategies may enhance patients’ perceived support resources and create a supportive environment for the sustained implementation of health-promoting behaviors. Third, enhancing and maintaining health activation should be central to interventions aimed at strengthening intrinsic behavioral motivation. Health activation may serve as a key motivational factor in the adoption and maintenance of health-promoting behaviors among patients with CHF. Nurses may use motivational interviewing, shared goal setting, and positive feedback to help patients clarify the meaning and value of health behaviors, thereby enhancing self-efficacy and willingness to act. Rather than setting overly ambitious goals at once, nurses should guide patients to adopt health-promoting behaviors gradually according to their disease condition and functional capacity, thereby reducing frustration and strengthening confidence in sustained behavioral change.

## 5 Conclusion

Health-promoting behaviors among patients with chronic heart failure were relatively low, particularly in the physical activity dimension, indicating insufficient engagement in long-term self-management. Health literacy, perceived social support, and health activation were significantly associated with health-promoting behaviors. Health activation partially mediated the associations between health literacy and health-promoting behaviors and between perceived social support and health-promoting behaviors. These findings suggest that improving knowledge and external support alone may be insufficient to sustain behavioral change. Strengthening patients’ intrinsic motivation and active participation in health management is also essential. Future interventions guided by the COM-B model should focus on enhancing health literacy, strengthening family and social support, and improving health activation. Such strategies may promote sustained health-promoting behaviors and improve long-term disease management in patients with chronic heart failure.

### 5.1 Limitation

This study has several limitations. First, the cross-sectional design allowed the identification of associations and statistical pathways between health-promoting behaviors and related factors among patients with CHF, but it limited the ability to infer longitudinal or causal relationships among variables. The mediation pathways identified in this study should therefore be understood as statistical associations consistent with the COM-B framework, rather than evidence of causal mechanisms. Future longitudinal or intervention studies are needed to further examine the dynamic changes and potential causal relationships among health literacy, perceived social support, health activation, and health-promoting behaviors.

Second, the participants were recruited using convenience sampling from only two tertiary hospitals in Shanghai, which may limit the representativeness and generalizability of the findings. Patients treated in tertiary hospitals in urban areas may have better access to medical resources, higher health literacy, and more structured disease-management support than those in rural areas or primary care settings. Therefore, rural populations and patients managed in community or primary care institutions were underrepresented in this study. In addition, because this study was conducted in a single urban region in China, the applicability of the findings to other regions, healthcare systems, and international populations remains uncertain. Future multicenter studies should include patients from rural areas, primary care settings, and diverse geographical and cultural contexts to improve the external validity of the findings.

Third, although the sample size met the recommended requirements for multivariable regression and path analysis, it remained relatively limited for complex machine-learning modeling. Therefore, the random forest analysis in this study was used only as an exploratory supplementary analysis to identify potentially important features rather than as a fully validated prediction model. Although the model showed acceptable predictive performance in the test set, possible overfitting cannot be fully excluded because of the limited sample size and the number of candidate predictors. The difference between the training and test R^2^ values may also suggest potential model optimism. In addition, repeated cross-validation, confidence intervals for performance metrics, and external validation were not conducted in the present study. Future studies with larger multicenter samples, repeated validation procedures, and independent external validation cohorts are needed to confirm the stability, reproducibility, and generalizability of the machine-learning findings. Furthermore, although the instruments used in this study have been validated in previous studies and showed acceptable internal consistency in the present sample, this study did not conduct a separate item-level confirmatory factor analysis or calculate composite reliability, average variance extracted, and discriminant validity for each latent construct. Therefore, the findings should be interpreted as path associations based on validated scale or dimension scores rather than as evidence from a fully validated latent measurement model. Future studies should further examine the measurement model and construct validity of these instruments in larger and more diverse CHF populations.

Fourth, all variables were measured using self-reported questionnaires, which may have introduced reporting bias, recall bias, social desirability bias, and common method variance. Although Harman’s single-factor test suggested that common method bias was unlikely to be severe, this test cannot definitively rule out common method variance. Future studies should consider multi-source data, objective behavioral indicators, and additional procedural or statistical controls to further reduce and assess common method bias. In addition, health literacy, perceived social support, and health activation are conceptually related constructs within the COM-B framework. Although the correlation matrix and multicollinearity diagnostics did not indicate serious statistical redundancy among these predictors, their conceptual overlap should be considered when interpreting the findings. Future studies may further examine the distinct and shared contributions of these constructs using longitudinal designs or more refined measurement models.

## Data Availability

The original contributions presented in the study are included in the article/Supplementary material, further inquiries can be directed to the corresponding author.
