# Supplementary material for: Health literacy, social support, and health activation as determinants of health-promoting behaviors in patients with chronic heart failure: a COM-B model–based path analysis
Source: Front Public Health. 2026 Jul 15;14:1895437. doi: 10.3389/fpubh.2026.1895437 (PMC13414931; doi:10.3389/fpubh.2026.1895437)
Supplement: Supplementary file 1 [file Data_Sheet_1.DOCX]

import pandas as pd

import numpy as np

import matplotlib.pyplot as plt

import warnings

import optuna

import shap

import scipy.stats as stats

import os

import joblib

import seaborn as sns

from sklearn.model_selection import train_test_split, cross_val_score

from sklearn.preprocessing import StandardScaler

from sklearn.ensemble import RandomForestRegressor

from sklearn import metrics

warnings.filterwarnings("ignore")

# 设置全局字体和符号显示（注册自定义中文字体）

from matplotlib.font_manager import fontManager

fontManager.addfont('/root/pfs_yangchen/learning/dz/SimHei.ttf')

plt.rcParams['font.family'] = 'SimHei'

plt.rcParams['axes.unicode_minus'] = False

# 全局 SHAP 可视化配置

# 可选方案: 'viridis', 'plasma', 'inferno', 'magma', 'cividis', 'seismic', 'coolwarm', 'bwr', 等

# 全局 SHAP 可视化配置

SHAP_COLORMAP = shap.plots.colors.red_blue

# 2. 数据加载与预处理

# -----------------------------------------------------------------------------

print("步骤 2: 数据加载与预处理...")

output_dir = 'rf'

os.makedirs(output_dir, exist_ok=True)

print(f"所有输出文件将保存在: {output_dir}\n")

try:

df = pd.read_excel('/root/pfs_yangchen/learning/dz/2026.2/2.28.1/修改一般资料数据分析-302_副本.xlsx')

except FileNotFoundError:

print("错误：未找到 文件。请检查路径。")

exit()

target_col = '健康素养总分'

features = df.columns.drop(target_col).tolist()

X = df[features]

y = df[target_col]

X_train, X_test, y_train, y_test = train_test_split(X, y, test_size=0.3, random_state=42)

scaler = StandardScaler()

X_train_scaled = scaler.fit_transform(X_train)

X_test_scaled = scaler.transform(X_test)

X_train_scaled = pd.DataFrame(X_train_scaled, columns=features)

X_test_scaled = pd.DataFrame(X_test_scaled, columns=features)

print("数据加载与预处理完成。\n")

# 3. 特征相关性热力图分析

# -----------------------------------------------------------------------------

print("步骤 3: 生成特征相关性热力图...")

plt.figure(figsize=(15, 12), dpi=300)

correlation_matrix = X.corr(method='pearson')

sns.heatmap(correlation_matrix, annot=True, fmt='.2f', cmap='coolwarm',

linewidths=.5, annot_kws={"size": 8})

plt.title('Feature Correlation Heatmap')

plt.xticks(rotation=45, ha='right')

plt.yticks(rotation=0)

plt.tight_layout()

plt.savefig(os.path.join(output_dir, 'feature_correlation_heatmap.pdf'), format='pdf')

plt.close()

print("特征相关性热力图已保存。\n")

# 4. Optuna 超参数优化

# -----------------------------------------------------------------------------

print("步骤 4: 使用 Optuna 进行超参数优化...")

def objective(trial):

n_estimators = trial.suggest_int('n_estimators', 100, 1000)

max_depth = trial.suggest_int('max_depth', 3, 20)

min_samples_split = trial.suggest_int('min_samples_split', 2, 20)

min_samples_leaf = trial.suggest_int('min_samples_leaf', 1, 10)

max_features = trial.suggest_categorical('max_features', ['sqrt', 'log2'])

model = RandomForestRegressor(

n_estimators=n_estimators,

max_depth=max_depth,

min_samples_split=min_samples_split,

min_samples_leaf=min_samples_leaf,

max_features=max_features,

random_state=42,

n_jobs=-1

)

score = cross_val_score(model, X_train_scaled, y_train, cv=3, scoring='neg_mean_squared_error')

return score.mean()

study = optuna.create_study(direction='maximize')

study.optimize(objective, n_trials=50)

print(f"找到的最佳超参数: {study.best_params}")

print(f"最佳交叉验证得分 (Negative MSE): {study.best_value}\n")

# 5. 最终模型训练、评估、保存与数据导出

# -----------------------------------------------------------------------------

print("步骤 5: 最终模型训练、评估、保存与数据导出...")

final_model = RandomForestRegressor(**study.best_params, random_state=42)

final_model.fit(X_train_scaled, y_train)

model_path = os.path.join(output_dir, 'rf_model.joblib')

joblib.dump(final_model, model_path)

print(f"最终模型已成功保存至: {model_path}")

y_pred_train = final_model.predict(X_train_scaled)

y_pred_test = final_model.predict(X_test_scaled)

metrics_train = {

"MSE": metrics.mean_squared_error(y_train, y_pred_train),

"RMSE": np.sqrt(metrics.mean_squared_error(y_train, y_pred_train)),

"MAE": metrics.mean_absolute_error(y_train, y_pred_train),

"R-squared": metrics.r2_score(y_train, y_pred_train)

}

metrics_test = {

"MSE": metrics.mean_squared_error(y_test, y_pred_test),

"RMSE": np.sqrt(metrics.mean_squared_error(y_test, y_pred_test)),

"MAE": metrics.mean_absolute_error(y_test, y_pred_test),

"R-squared": metrics.r2_score(y_test, y_pred_test)

}

print("\n性能指标:")

print(pd.DataFrame({'Train': metrics_train, 'Test': metrics_test}))

performance_df = pd.DataFrame({'Train': metrics_train, 'Test': metrics_test})

performance_path = os.path.join(output_dir, 'model_performance_metrics.xlsx')

performance_df.to_excel(performance_path, index=True)

print(f"\n模型性能指标已导出至: {performance_path}")

predictions_df = pd.DataFrame({'Actual_Value': y_test, 'Predicted_Value': y_pred_test})

predictions_path = os.path.join(output_dir, 'predictions_vs_actuals.xlsx')

predictions_df.to_excel(predictions_path, index=False)

print(f"预测值与真实值对比已导出至: {predictions_path}\n")

# 6. 特征重要性分析与导出

# -----------------------------------------------------------------------------

print("步骤 6: 特征重要性分析与导出...")

importances = final_model.feature_importances_

native_importance_df = pd.DataFrame({

'Feature': features,

'Importance': importances

}).sort_values(by='Importance', ascending=False)

native_importance_path = os.path.join(output_dir, 'model_native_importance.xlsx')

native_importance_df.to_excel(native_importance_path, index=False)

print(f"模型自带的重要性已导出至: {native_importance_path}")

plt.figure(figsize=(10, 6), dpi=300)

plt.barh(native_importance_df.sort_values(by='Importance')['Feature'],

native_importance_df.sort_values(by='Importance')['Importance'], color='skyblue')

plt.xlabel('Importance')

plt.title('Random Forest - Built-in Feature Importance')

plt.tight_layout()

plt.savefig(os.path.join(output_dir, 'rf_builtin_importance.pdf'), format='pdf')

plt.close()

print("\n计算 SHAP 值 (这可能需要一些时间)...")

explainer = shap.TreeExplainer(final_model)

shap_values = explainer.shap_values(X_test_scaled)

print("SHAP 值计算完成。")

shap_contribution_df = pd.DataFrame(shap_values, columns=features)

shap_contribution_path = os.path.join(output_dir, 'shap_contribution_values.xlsx')

shap_contribution_df.to_excel(shap_contribution_path, index=True)

print(f"SHAP 详细贡献值已导出至: {shap_contribution_path}")

shap_global_importance_df = pd.DataFrame({

'Feature': features,

'Avg_Abs_SHAP_Value': np.abs(shap_values).mean(axis=0)

}).sort_values(by='Avg_Abs_SHAP_Value', ascending=False)

shap_global_path = os.path.join(output_dir, 'shap_global_importance.xlsx')

shap_global_importance_df.to_excel(shap_global_path, index=False)

print(f"SHAP 全局重要性已导出至: {shap_global_path}")

print("\n正在生成并保存全套 SHAP 图...")

shap.summary_plot(shap_values, X_test_scaled, plot_type="dot", show=False, cmap=SHAP_COLORMAP)

plt.title("SHAP Summary Plot")

plt.savefig(os.path.join(output_dir, 'rf_shap_summary.pdf'), format='pdf', bbox_inches='tight')

plt.close()

shap.summary_plot(shap_values, X_test_scaled, plot_type="bar", show=False)

plt.title("SHAP Global Feature Importance")

plt.savefig(os.path.join(output_dir, 'rf_shap_bar.pdf'), format='pdf', bbox_inches='tight')

plt.close()

print(" - 摘要图和条形图已保存。")

top_features = shap_global_importance_df['Feature'].head(30).tolist()

for feature in top_features:

shap.dependence_plot(feature, shap_values, X_test_scaled, show=False, cmap=SHAP_COLORMAP)

plt.title(f"SHAP Dependence Plot for {feature}")

# 添加y=0的水平虚线

plt.axhline(y=0, linestyle='--', color='gray', alpha=0.7)

plt.savefig(os.path.join(output_dir, f'rf_shap_dependence_{feature}.pdf'), format='pdf', bbox_inches='tight')

plt.close()

print(f" - 依赖图已保存 (针对特征: {', '.join(top_features)})。")

try:

num_samples_for_heatmap = min(500, len(X_test_scaled))

shap_explanation = shap.Explanation(

values=shap_values[:num_samples_for_heatmap, :],

base_values=explainer.expected_value,

data=X_test_scaled.iloc[:num_samples_for_heatmap, :],

feature_names=features

)

shap.plots.heatmap(shap_explanation, show=False, cmap=SHAP_COLORMAP)

plt.title("SHAP Heatmap Plot")

plt.savefig(os.path.join(output_dir, 'rf_shap_heatmap.pdf'), format='pdf', bbox_inches='tight')

plt.close()

print(" - SHAP 热力图已保存。")

except Exception as e:

print(f" - 未能生成 SHAP 热力图。错误: {e}")

for i in range(3):

shap.force_plot(

np.round(explainer.expected_value, 2),

np.round(shap_values[i,:], 2),

np.round(X_test_scaled.iloc[i,:], 2),

show=False, matplotlib=True

)

plt.title(f"SHAP Force Plot for Sample {i}")

plt.savefig(os.path.join(output_dir, f'rf_shap_force_plot_sample_{i}.pdf'), format='pdf', bbox_inches='tight')

plt.close()

print(" - 单样本力图已保存 (前 3 个样本)。")

print("\n计算 SHAP 交互值 (警告: 此过程可能非常缓慢)...")

shap_interaction_values = explainer.shap_interaction_values(X_test_scaled)

print("SHAP 交互值计算完成。")

shap.summary_plot(shap_interaction_values, X_test_scaled, show=False, cmap=SHAP_COLORMAP)

plt.title("SHAP Interaction Values Summary Plot")

plt.savefig(os.path.join(output_dir, 'rf_shap_interaction.pdf'), format='pdf', bbox_inches='tight')

plt.close()

print(" - 交互作用图已保存。\n")

# 7. 高级散点图可视化

# -----------------------------------------------------------------------------

print("步骤 7: 生成高级散点图...")

r2_train, mae_train = metrics_train['R-squared'], metrics_train['MAE']

r2_test, mae_test = metrics_test['R-squared'], metrics_test['MAE']

scale_factor, confidence = 1.5, 0.95

z_train = np.polyfit(y_train, y_pred_train, 1)

p_train = np.poly1d(z_train)

x_extended_train = np.linspace(min(y_train), max(y_train), 100)

predicted_extended_train = p_train(x_extended_train)

residuals_train = y_pred_train - p_train(y_train)

mean_error_train = np.mean(residuals_train**2)

t_value_train = stats.t.ppf((1 + confidence) / 2., len(y_train) - 1)

ci_extended_train = t_value_train * scale_factor * np.sqrt(mean_error_train) * np.sqrt(1 / len(y_train) + (x_extended_train - np.mean(y_train))**2 / np.sum((y_train - np.mean(y_train))**2))

z_test = np.polyfit(y_test, y_pred_test, 1)

p_test = np.poly1d(z_test)

x_extended_test = np.linspace(min(y_test), max(y_test), 100)

predicted_extended_test = p_test(x_extended_test)

residuals_test = y_pred_test - p_test(y_test)

mean_error_test = np.mean(residuals_test**2)

t_value_test = stats.t.ppf((1 + confidence) / 2., len(y_test) - 1)

ci_extended_test = t_value_test * scale_factor * np.sqrt(mean_error_test) * np.sqrt(1 / len(y_test) + (x_extended_test - np.mean(y_test))**2 / np.sum((y_test - np.mean(y_test))**2))

train_color, test_color = '#1f77b4', '#ff7f0e'

confidence_train_color, confidence_test_color = '#aec7e8', '#ffbb78'

fig = plt.figure(figsize=(10, 8), dpi=600)

gs = fig.add_gridspec(4, 4, hspace=0.3, wspace=0.3)

ax_main = fig.add_subplot(gs[1:, :-1])

ax_hist_x = fig.add_subplot(gs[0, :-1], sharex=ax_main)

ax_hist_y = fig.add_subplot(gs[1:, -1], sharey=ax_main)

ax_main.scatter(y_train, y_pred_train, color=train_color, label="Training Predicted Values", alpha=0.6)

ax_main.plot(x_extended_train, predicted_extended_train, color=train_color, alpha=0.9, label=f"Training Line of Best Fit\n$R^2$ = {r2_train:.2f}, MAE = {mae_train:.2f}")

ax_main.fill_between(x_extended_train, predicted_extended_train - ci_extended_train, predicted_extended_train + ci_extended_train, color=confidence_train_color, alpha=0.5, label="Training 95% Confidence Interval")

ax_main.scatter(y_test, y_pred_test, color=test_color, label="Testing Predicted Values", alpha=0.6)

ax_main.plot(x_extended_test, predicted_extended_test, color=test_color, alpha=0.9, label=f"Testing Line of Best Fit\n$R^2$ = {r2_test:.2f}, MAE = {mae_test:.2f}")

ax_main.fill_between(x_extended_test, predicted_extended_test - ci_extended_test, predicted_extended_test + ci_extended_test, color=confidence_test_color, alpha=0.5, label="Testing 95% Confidence Interval")

ax_main.plot([min(y_train.min(), y_test.min()), max(y_train.max(), y_test.max())], [min(y_train.min(), y_test.min()), max(y_train.max(), y_test.max())], color='grey', linestyle='--', alpha=0.6, label="1:1 Line")

ax_main.set_xlabel("Observed Values", fontsize=12)

ax_main.set_ylabel("Predicted Values", fontsize=12)

ax_main.legend(loc="upper left", fontsize=10)

ax_hist_x.hist(y_train, bins=20, color=train_color, alpha=0.7, edgecolor='black', label="Training Observed")

ax_hist_x.hist(y_test, bins=20, color=test_color, alpha=0.7, edgecolor='black', label="Testing Observed")

ax_hist_x.tick_params(labelbottom=False)

ax_hist_x.legend(loc="upper right", fontsize=8)

ax_hist_y.hist(y_pred_train, bins=20, orientation='horizontal', color=train_color, alpha=0.7, edgecolor='black')

ax_hist_y.hist(y_pred_test, bins=20, orientation='horizontal', color=test_color, alpha=0.7, edgecolor='black')

ax_hist_y.tick_params(labelleft=False)

fig.suptitle("Random Forest: Observed vs. Predicted Values", fontsize=16)

plt.tight_layout(rect=[0, 0, 1, 0.96])

plt.savefig(os.path.join(output_dir, 'rf_scatterplot.pdf'), format='pdf', bbox_inches='tight')

plt.close()

print("高级散点图生成完成。")

print(f"\n所有任务已完成！模型文件、图表和 Excel 数据已全部保存至 {output_dir}")
